# The State of Lupus Clinical Trials: Minority Participation Needed

**DOI:** 10.3390/jcm8081245

**Published:** 2019-08-17

**Authors:** Saira Z. Sheikh, Nicole I. Wanty, Joncel Stephens, Kristen D. Holtz, Sheryl McCalla

**Affiliations:** 1 UNC Thurston Arthritis Research Center, Chapel Hill, NC 27599, USA; 2 Department of Medicine, Division of Rheumatology, Allergy and Immunology, University of North Carolina at Chapel Hill School of Medicine, Chapel Hill, NC 27599, USA; 3 KDH Research and Communication, Atlanta, GA 30361, USA; 4 Strategic Initiatives, American College of Rheumatology, Atlanta, GA 30319, USA

**Keywords:** lupus, lupus health disparities, clinical trials, clinical trial disparities, clinical trial diversity

## Abstract

In the United States, the reported prevalence of lupus is 100,000 to 500,000 patients. Lupus disproportionately affects minority populations, including African Americans and Latinos, and the associated health disparities are substantial. Women are at a higher risk of lupus than men and lupus prevalence is the highest in African Americans and Latinos compared to non-Hispanic whites. African Americans and Latinos also have increased disease symptom severity, experience more lupus-related complications, and have a two- to three-fold mortality rate compared to non-Hispanic Whites. Lupus clinical trials offer opportunities for quality care and can result in new treatment options, but African Americans and Latinos are underrepresented in clinical trials because of substantial patient- and provider-side barriers. In conjunction with the limited knowledge of clinical trials that potential participants may have, the healthcare staff approaching participants have limited time to adequately educate and explain the aspects of clinical trials. Indeed, ninety percent of clinical trials fail to meet their recruitment goals on time, so a multi-faceted approach is necessary to address the issue of low minority participation in clinical trials.

## 1. Introduction

Lupus disproportionately affects minority populations, including African Americans and Latinos. In the United States (U.S.), the reported prevalence of systemic lupus erythematosus (hereinafter “lupus”) is 100,000 to 500,000 patients [1,2]. Lupus is an autoimmune disorder that causes inflammation and affects the skin, joints, and multiple organ systems in the body [3]. Lupus symptoms vary widely, ranging from mild to severe [3,4,5], and can be associated with dermatologic, renal, and other manifestations. Epidemiologic studies of these manifestations are rare, because there is controversy as to whether these represent different spectra of the same disease or are distinct disease phenotypes [6]. Studies have found that the incidence of cutaneous forms of lupus is comparable to that of the published incidence of systemic lupus, at about four new cases per 100,000 persons per year in the U.S. [7]. However, published studies are limited in their ability to draw conclusions in how cutaneous forms affect different racial or ethnic groups due to study populations not being representative of the U.S. [8]. Typically, with lupus, patients experience fatigue, rashes, arthritis, and fever [5], but they may also experience serious, life-threatening symptoms, including chronic inflammation and damage to the kidneys, heart, lungs, eyes, and brain [2,3]. There is no cure for lupus [9] and cutaneous lupus can result in permanent and disfiguring effects or irreversible damage to the skin. Clinical trials offer promise and potentially new therapeutic options for patients living with aggressive dermatologic involvement in lupus. Early diagnosis and proper treatment reduces lupus morbidity and mortality [10], allowing patients diagnosed with lupus to reduce the physical, mental, and social effects of lupus; decrease possible medical complications; and decrease their risk of death [10].

There are substantial disparities in clinical trial participation. Clinical trials systematically explore new and better treatments for diseases. Despite the fact that there are approximately 100,000 clinical trials operating in the United States at any given time [11], minority participation in these trials falls well short of a representative proportion of the population. African Americans represent approximately 12 percent of the U.S. population, but only 5 percent of clinical trial participants [12]. Similarly, Latinos make up 16 percent of the U.S. population, but only 1 percent of clinical trial research participants [12].

Minority participation in lupus clinical trials is low. Low minority participation in lupus clinical trials results in a lack of data on the effectiveness, safety, and side effects of treatment within populations with the highest incidence, prevalence, morbidity, and mortality [13]. There are approximately 400 sites currently seeking participation in lupus clinical trials in the United States and Canada [11]. However, clinical trials for lupus have had limited success in recruiting minority participants. New biologic therapies present an alternative to conventional immunosuppressant therapies and have the potential to reduce the dependence on corticosteroids [12]. In 2011, belimumab (Benlysta) became the first drug approved by the U.S. Food and Drug Administration (FDA) for lupus since 1955 [14]. However, despite lupus’ disproportionate prevalence among African Americans and Latinos, the belimumab clinical trials consisted of only 14 percent African American and 20 percent Latino; the small sample size led to inconclusive results on the effectiveness of belimumab for African Americans and Latinos [14,15]. Therefore, Human Genome Sciences and GlaxoSmithKline conducted an international belimumab Phase IV clinical trial specifically with African Americans and other patients of Black race [14,16]. This clinical trial enrolled 503 patients and was completed in 2019 [16]. In a recent review of national and international randomized controlled trials (RCTs) with lupus patients from 1997 to 2017, researchers compared lupus prevalence and representation in RCTs by race/ethnicity [17]. Researchers calculated that Blacks make up 43 percent of lupus cases, but only 14 percent of RCT enrollees. Additionally, researchers noted that Black representation in RCTs in the U.S. has decreased between 2006 and 2011. Researchers also noted that the inclusion of Latinos in RCTs has increased over time to 16 percent of lupus cases and 21 percent of RCT enrollees, but new sites in Latin American countries may have contributed to this increase [17].

Minority participation in lupus clinical trials is essential to reduce lupus health disparities at the individual and systems level. Indirect and direct costs of health disparities result in economic losses of $309 billion per year and approximately 30 percent of direct medical costs for minorities are excess costs because of health disparities [13]. Clinical trials can reduce health disparities because there is evidence that patients in clinical trials have better outcomes than patients not enrolled in trials—even those in the control group [18]. What is more, diverse study populations for clinical trials can improve the generalization of findings on the efficacy, side effects, and risk of medications, helping patients make better treatment decisions with their provider and improving health outcomes overall.

## 2. Addressing the Problem by Increasing Minority Participation

There are a myriad of barriers to minority participation in lupus clinical trials. The barriers to minority participation in clinical trials are two-sided, including patient-side barriers to participation and provider-side barriers to referring patients. In Table 1, we provide a brief list of patient-side barriers shared by African American and Latino patients and in Table 2, we provide barriers that providers may encounter in referring patients to clinical trials.

Providers, writ large, play an important role in increasing minority participation in lupus clinical trials. Most patients (77%) who participate in a clinical trial learn about it from their provider [35], yet providers refer less than one percent of their patients every year to clinical trials and almost half of providers report not knowing where to refer patients for clinical trials [33]. Initiatives to increase the referral of minority patients to lupus clinical trials should include rheumatologists and other specialists, primary care providers (PCPs), other healthcare professionals, and paraprofessionals [36]. Research suggests that integrating multiple staff into the recruitment effort creates a “supportive infrastructure [36]” that increases successful patient enrollment into clinical trials [36]. Nurses [37] and physician assistants [38] spend more time with patients compared to physicians, and may have varying levels of influence on patient recruitment into clinical trials [33]. Nurses are considered a “trusted source of information about clinical trials [33],” and nearly 70 percent report feeling comfortable introducing their patients to clinical trials [33] and many are as effective at clinical trial recruitment as external patient navigators [36].

Patient support and education outside of the doctor–patient relationship is essential to increase minority participation in lupus clinical trials. Outside of the provider–patient interaction, patient advocates can help mitigate many of the barriers identified in Table 1 by providing peer education and support to other patients with lupus. Patient advocacy has been described broadly as acting on a patient’s unmet needs, which may include informing, protecting, and speaking for patients [39]. Patient advocates have a broad role [40] and are defined in many ways as peers who (1) defend the rights of property and other [41]; (2) help guide a patient through the screening, diagnosis, treatment, and follow-up of a medical condition [42]; and (3) help patients communicate with their healthcare providers so they get the information they need to make decisions [42]. Patient advocates are beneficial to clinical trials’ referrals, enrollment, and retention [43]. In fact, the Institute of Medicine recently called for the greater involvement of patient advocates in the design of clinical trials and in patient recruitment efforts to encourage patient acceptance and participation in clinical trials [44]. On the same token, community health workers (CHWs), also known as *promotores de salud*, and many other titles, are frontline personnel who provide education and connect clients to services by helping them overcome barriers in their communities and health systems [45]. CHWs are trusted members of their often diverse, multicultural communities, which allows them to build trust between their community and the health systems [46]. Overall, using CHWs increases clinical trial enrollment by minority populations [47,48], and decreases clinical trial refusal by minority populations [45]. Varied recruitment strategies for different clinical trials and target populations make it difficult to compare the effectiveness of any single recruitment strategy., However, the clinical trials that have evaluated the effect of community outreach, including that of CHWs, on recruitment encourages further exploration of this strategy [49].

There is a need for multifaceted approaches for increasing minority participation in clinical trials. When individuals who declined clinical trial participation were probed with questions on why they declined, one participant explained, “I had no idea about clinical trials. It was foreign to me. It’s like a foreign word. I had never heard of it before [50].” With the limited time that providers have with patients during medical visits, educating providers on methods to quickly and effectively deliver the most helpful information for a minority patient is important, as is providing additional patient support so that they are able to understand and make informed decisions about clinical trial participation. Lying at the center of a network of providers referring minority patients to clinical trials is education and starting the clinical trial conversation. It is imperative that providers are equipped to discuss local clinical trials and to answer patient questions or concerns about clinical trials. A study exploring how awareness changes attitudes toward clinical trials and the benefits of participation found that 85 percent of patients were either unaware or unsure that participation in a clinical trial was an option at the time of diagnosis. Furthermore, 75 percent of these patients said they would have been willing to enroll had they known it were possible. In Table 3, we provide a brief list of categories in which interventions can address the issue of low minority enrollment in clinical trials and programs developed or being developed within these categories.

## 3. Future Directions

In summary, a multifaceted approach to interventions to increase minority participation in clinical trials is needed. An effective intervention can employ various methods and target various audiences, but it is important that the intervention is appropriate for the recruitment goals of the clinical trial and appropriate for the audience of healthcare providers and professionals or patients receiving the intervention. The literature on best practices to support multifaceted efforts to recruit patients into clinical trials indicates that there are characteristics that generally lead to more effective intervention programs, as follows:

(1) Online delivery of training courses and information to patients and providers. These methods are low-cost and preferred by healthcare providers and professionals, as well as patients [24]. Training courses centered around educating patients on clinical trials can give healthcare providers and professionals an opportunity to advance their education in healthcare delivery and medical practice (e.g., Continuing Medical Education credits, Continuing Education Credits, etc.). Efforts for patient-centered education on clinical trials exist in the form of delivering engaging, evidence-based and patient-centered content. There is a strong empirical foundation for best practices in the delivery of health-related content via mobile applications and technologies. Interactive media, such as video games, simulators, eLearning, and mobile apps, can also improve patient comprehension of research study protocols and risks, patient decision-making about clinical trials, and patient engagement throughout clinical trials [32];
(2) Culturally and linguistically appropriate patient materials for patients and providers to use. Clinical trial education programs are most effective for patients when they are culturally and linguistically appropriate, less technical, and more personalized [51,52,53,54]. For healthcare providers and professionals, culturally and linguistically appropriate patient materials help them easily inform patients about lupus clinical trials. Furthermore, the use of effective supplemental materials in patient education has been shown to increase the likelihood of providers following through with a recommended behavior (i.e., referring lupus patients to lupus clinical trials) [55];
(3) Active dissemination methods for providers and patients. Active dissemination methods such as outreach conducted by healthcare professionals to other healthcare professionals, are more effective than passive dissemination such as mail, e-mails, or newsletters [56,57]. The same goes for patients, as patient advocates have been shown to help mitigate many of the barriers identified in Table 1 by providing peer education and support to other patients with lupus. Patient advocates are beneficial to clinical trials’ referrals, enrollment, and retention [43]. In fact, the Institute of Medicine recently called for the greater involvement of patient advocates in the design of clinical trials and in patient recruitment efforts to encourage patient acceptance and participation in clinical trials [44]. For healthcare providers and professionals, colleagues are often the main source of information about clinical trials [58]. Studies find that healthcare providers and professionals who are confident in the principal investigators or site conducting a clinical trial, and who are familiar with the trial’s protocols, are much more likely to refer patients [33].

Efforts to develop multifaceted interventions that address provider and patient-side barriers to clinical trial participation are ongoing. The recruitment of patients plays an important role in the development of new therapies for lupus. From basic science to potential treatments, understanding disparities in clinical trial participation and addressing these disparities improves the ability to target and recruit from patient populations in which the disease is more prevalent and the disease burden is the highest.

## Figures and Tables

**Table 1:** Patient-side clinical trial participation barriers.

Barrier	Example
Access	Lack of access to rheumatologists who are most likely to know about lupus clinical trials [19] Lack of transportation to the clinical trial site [20], child care [21], or paid leave to miss work [22] Lack of health insurance and cost concerns [20] ^1^ Lack of bilingual research staff or informational material about clinical trials [22]
Opportunity	Lack of awareness of clinical trials [23] Lack of minority providers to refer minority patients to clinical trials [24] General lack of referral by providers ^2^
Mistrust	A history of exploitation in research in African American communities [20,23,25] Fear among Latino immigrants of being deported as a result of participating in a clinical trial [22] Feelings of uncertainty and anxiety about clinical trials [20] Fear of a negative impact on quality of life through loss of autonomy and side effects [24,26] Discomfort with “experiments” and feeling like a “guinea pig” [20,23]
Health literacy	Lack of education about disease [27] A dislike and misunderstanding of study group randomization [28] Feelings of intimidation and misunderstanding about the informed consent process [28] Belief that the clinical trial has little or no benefits [22,28] Challenges understanding study documentation written at too high a reading level [29]
Cultural	Excluding family members in enrollment and participation deters Latino patients [30] Lack of a friendly patient–provider relationship [31]

^1^ Many patients are unaware that many clinical trials offer free or reduced-cost care [32]. ^2^ More than three-fourths of patients state they would have been willing to participate if presented with the opportunity [23].

**Table 2:** Provider-side clinical trial recruitment barriers.

Barrier	Example
Awareness and knowledge	Lack of access to clinical trial information [33] Lack of familiarity with clinical trial sites and principal investigators [33] Lack of knowledge about the clinical trial protocol [33] Disconnect between the patient’s eligibility and provider assessment of patient’s eligibility [33]
Attitude/implicit bias	Beliefs that minority patients will not understand and adhere to specific protocols [34] Beliefs that the clinical trial could have a negative impact on the provider–patient relationship [20] (if they refer their patient to a clinical trial, they will lose their patient to the clinical trial’s practice or it will have a negative effect on their interpersonal relationship [20]) Beliefs that the clinical trial is unsafe or coercive [20]
Logistical	Lack of time to talk to patients and to learn about clinical trials [33] Lack of connection with clinical trial site or principal investigator [33] Lack of clinical trials in close proximity to the provider’s office [33]

**Table 3:** Interventions.

Intervention Category	Example
Provider-focused education interventions	Materials to Increase Minority Involvement in Clinical Trials (MIMICT) An education program for specialists (rheumatologists, nephrologists, dermatologists, and others), primary care physicians, RNs, NPs, and PAs. Developed by the American College of Rheumatology, with funding from the US Department of Health and Human Services Office of Minority Health.
Partnership-based interventions	Community Health Worker (CHW) Lupus Clinical Trials Training (LuCTT) A program to develop community partnerships and train CHWs to support the recruitment and enrollment of minority people with lupus into clinical trials, developed by the American College of Rheumatology, with funding from the US Department of Health and Human Services Office of Minority Health.
Patient-mediated interventions	Programs to address Unmet needs and promote Representation of all Participants in Lupus clinical trials using mobile technology for Engagement (PURPLE) A patient-focused digital tool delivering culturally and linguistically appropriate patient clinical trial education, developed by University of North Carolina Thurston Arthritis Research Center, with funding from the Fund for Excellence in Lupus and Sjogren’s.
Peer-mediated interventions	Patient Advocates for Lupus Studies (PALS) Peer-to-peer education program in which individuals living with lupus who have participated in clinical research activities provide education and guidance to those living with lupus who have never participated, developed by Lupus Therapeutics in partnership with KDH Research and Communication.
